# Dual roles of innate immune cells and cytokines in shaping the breast cancer microenvironment

**DOI:** 10.3389/fimmu.2025.1654947

**Published:** 2025-08-20

**Authors:** Chen Zhang, Wei Liu, Ping Yang, Rubing Lin, Lulan Pu, Hongying Zhang

**Affiliations:** ^1^ Department of General Surgery, Wusheng County People’s Hospital (Wusheng Hospital Affiliated Hospital of North Sichuan Medical College), Guangan, China; ^2^ Department of Clinical Medicine, North Sichuan Medical College, Nanchong, China; ^3^ Department of General Surgery, Nanbu County People’s Hospital, Nanchong, China; ^4^ Department of Orthopedics, Shenzhen Children’s Hospital, Shenzhen, China; ^5^ Department of Anatomy, North Sichuan Medical College, Nanchong, China; ^6^ Department of Thyroid and Breast Surgery, Gaoping District People’s Hospital of Nanchong City (Affiliated Hospital of China West Normal University), Nanchong, China

**Keywords:** breast cancer, tumor immune microenvironment, innate immune cells, immunomodulatory factors, immune surveillance, prognostic biomarkers

## Abstract

Breast cancer remains the most frequently diagnosed malignancy and a leading cause of cancer-related mortality among women worldwide. Increasing evidence underscores the pivotal yet paradoxical roles of innate immune cells and their associated cytokines in orchestrating the dynamic landscape of the breast tumor immune microenvironment (TIME). Innate immune effectors, including tumor-associated macrophages (TAMs) and natural killer (NK) cells, exert dual functions by either initiating robust antitumor responses or facilitating immune evasion, metastatic dissemination, and therapeutic resistance. For instance, MDSCs suppress T and NK cell activity via STAT3/NF-κB signaling and modulate the cytokine milieu through IL-10 and TGF-β. Similarly, M2-polarized TAMs promote angiogenesis, epithelial–mesenchymal transition, and chemoresistance via IL-10/STAT3/Bcl-2 pathways. In contrast, NK cells and CD103^+^ DCs mediate tumor cell cytolysis and prime antigen-specific immunity, though their activity is frequently compromised in advanced disease. Moreover, key cytokines and chemokines, including IL-6, IL-10, IL-8, TNF-α, TGF-β, and CCL2/5, demonstrate subtype-specific and context-dependent effects, acting as both tumor-promoting and tumor-suppressing agents through complex signaling networks. This review highlights the dualistic nature of innate immune components in breast cancer, discusses their prognostic and therapeutic implications, and proposes novel intervention strategies, such as TAM repolarization, and cytokine modulation, to reprogram the TIME and restore effective immune surveillance, particularly in aggressive subtypes like triple-negative breast cancer.

## 1 Introduction

Breast cancer is the most prevalent malignancy and primary cause of cancer-related death in women worldwide (1, 2). Recent advances in immunotherapy have shown significant potential in improving treatment outcomes and survival rates (3, 4). A comprehensive analysis of the tumor immune microenvironment could optimize immunotherapeutic approaches for breast cancer (5). Studies indicate that immune cells and mediators within this microenvironment not only combat tumors but also promote immune evasion, facilitating cancer progression (6, 7). Shared signaling pathways between immune and oncogenic processes regulate cell proliferation, apoptosis, and angiogenesis (8–11). Early in tumor development, malignant cells manipulate immune components to avoid detection, while advanced tumors establish an immunosuppressive niche, resisting immune-mediated destruction (12).

Effector T cells play a critical role in antitumor immunity, yet their function is often suppressed in breast cancer (13). Meanwhile, innate immune cells including macrophages, natural killer (NK) cells, and myeloid-derived suppressor cells (MDSCs), and mediators exhibit remarkable functional plasticity, exerting either tumoricidal or tumor-promoting effects depending on microenvironmental cues (14–16). The diversity of these immune components varies by tumor subtype and stage, offering diagnostic and prognostic value (17, 18). This review explores the mechanisms of innate immune cells and mediators in breast cancer progression, highlighting their clinical implications.

## 2 Innate immune cells in the breast cancer immune microenvironment

### 2.1 NK cells mediate direct antitumor cytotoxicity

NK cells are glycolipid-reactive lymphocytes with intrinsic cytotoxic capacity against tumor cells. Studies have demonstrated that activation of NK cells enhances antitumor immunity and survival in murine models of postoperative metastatic breast cancer (19, 20). Chemotherapeutic agents such as gemcitabine and cyclophosphamide may facilitate NK cell recruitment to the primary tumor site, and in combination with NK cell activation, significantly improve antitumor efficacy and reduce recurrence rates (21–23). In HER2-positive patients receiving adjuvant chemotherapy, the tumor microenvironment exhibited increased infiltration of NK cells and regulatory T cells, with this population showing reduced chemotherapy-related pathological responses (24–26). The increase in regulatory T cells may be associated with NK cell-mediated inhibition of tumor stem cell proliferation, reversal of MDSC-induced immunosuppression, and restoration of T cell proliferation (27, 28). Moreover, distinct NK cell subsets are associated with different stages of breast cancer progression. For instance, CD56^bright^CD16^+^ and CD56^dim^CD16^−^ NK cell populations are significantly elevated in the peripheral blood of patients with progressive invasive breast cancer (29). Intratumoral CD56^+^ NK cell density is positively correlated with tumor grade and stage, and although a lower level of CD56^+^ NK cells is generally indicative of favorable prognosis, no clear association has been established with overall survival (30). However, the cytotoxic activity of NK cells is often impaired in advanced-stage breast cancer due to multiple tumor-induced immunosuppressive mechanisms (31). Notably, transforming growth factor-beta (TGF-β), abundantly present in the tumor microenvironment, downregulates the expression of key NK cell–activating receptors such as NKG2D and NKp30, thereby compromising tumor cell recognition and cytolytic function (32–36). Additionally, MDSCs inhibit NK cell cytotoxicity by producing reactive oxygen species, particularly hydrogen peroxide, and immunosuppressive cytokines like TGF-β, which further dampen NK cell activation and IFN-γ production (37, 38). These suppressive pathways collectively lead to NK cell exhaustion, reduced granzyme B/perforin secretion, and impaired tumor control (39, 40). Understanding the mechanisms behind their functional impairment, particularly receptor downregulation and MDSC-mediated suppression, may yield valuable insights for diagnostic and therapeutic innovation.

### 2.2 Dendritic cells present tumor antigens to activate antigen-specific T cells

DCs, as pivotal antigen-presenting cells in adaptive immunity, play a central role in antitumor responses by promoting the expression of both exogenous and endogenous major histocompatibility complex (MHC) class I and II molecules (41, 42). They facilitate tumor antigen trafficking to draining lymph nodes, cross-present antigens to activate cytotoxic T lymphocytes (CTLs), and orchestrate T cell differentiation and activation (43–45). Among DC subsets, CD103^+^ conventional type 1 dendritic cells (cDC1s) are uniquely equipped for antigen cross-presentation, a process by which exogenous tumor-derived antigens are processed and presented on MHC class I molecules (46, 47). This activation relies on key components such as the Sec22b vesicle trafficking protein, the BATF3 transcription factor, and cross-priming signals via the STING and type I interferon pathways (48–50). Upon migration to lymph nodes, CD103^+^ DCs engage CD8^+^ T cells through MHC-I–peptide complexes and co-stimulatory molecules such as CD80/CD86, ultimately inducing tumor-specific cytotoxic responses (51). However, the frequency and functional competence of CD103^+^ DCs are often reduced in advanced breast cancer, leading to impaired priming of effector CD8^+^ T cells (52, 53). Tumor-derived suppressive cytokines such as IL-10, and TGF-β, as well as hypoxic conditions, inhibit CD103^+^ DC differentiation and antigen-presenting capacity (54, 55). Additionally, elevated expression of PD-L1 on dysfunctional DCs can further suppress T cell activation. These alterations in CD103^+^ DC function contribute to ineffective antitumor immunity, enhanced immune evasion, and poor therapeutic outcomes. Recent studies have shown that CD103^+^ DCs are capable of delivering intact tumor antigens to peripheral lymph nodes, thereby priming tumor-specific CD8^+^ T cells and locally suppressing PD-L1 activity (52, 56). However, current research on DCs in the breast cancer context remains limited, and further mechanistic studies are warranted.

### 2.3 MDSCs promote breast cancer progression and affect prognosis through multiple signaling pathways and immunomodulatory factors

MDSCs comprise a heterogeneous population of myeloid progenitor cells, including immature granulocytic (G-MDSC) and monocytic (M-MDSC) subsets (57, 58). Clinical data indicate a close association between MDSC levels and breast cancer stage, tumor burden in metastatic disease, and chemotherapy efficacy (59). Elevated MDSC levels are linked to increased risk of postoperative recurrence and metastasis, whereas patients with lower MDSC counts demonstrate higher rates of pathological complete response (60, 61). In stage IV breast cancer patients, high levels of HLA-DR^neg/low^, CD33^+^, CD11b^+^ MDSCs are associated with significantly reduced survival (62). Furthermore, MDSCs can promote the production of IL-1β and IL-17, reducing the efficacy of chemotherapeutic agents such as 5-fluorouracil and gemcitabine, thereby adversely affecting prognosis (63, 64). Mechanistically, MDSCs in the breast tumor microenvironment promote invasion and metastasis via pathways such as STAT3-NF-κB-IDO, STAT3/IRF-8, and PTEN/Akt (65), involving both inhibitory and stimulatory cytokines. These pathways drive MDSC expansion and lead to downstream functional consequences that impair antitumor immunity. For instance, activation of STAT3 induces expression of arginase-1 and iNOS, resulting in depletion of L-arginine and accumulation of reactive oxygen species (ROS), which in turn inhibit CD8^+^ T cell receptor ζ-chain expression and induce T cell anergy (66–68). Concurrently, IL-2 production suppression further impairs T cell proliferation and effector function (69). The PTEN/AKT axis supports MDSC resistance to apoptosis and enhances their immunosuppressive capacity through sustained IL-10 and TGF-β secretion (65). Collectively, these mechanisms contribute to immune evasion, tumor progression, and treatment resistance.

On one hand, cytokines such as TGF-β and Flt3L induce CD11b^+^ MDSC differentiation, while IL-6 and IL-18 promote CD33^+^ MDSC proliferation (70). Chemokines including CXCL5/CXCR2 are essential for MDSC recruitment in 4T1 BALB/c murine tumor models, while CCL1, CCL2, CCL5, GM-CSF, and G-CSF facilitate MDSC expansion and aggregation in the tumor milieu (71–73). On the other hand, MDSCs suppress antitumor immune responses by modulating the cytokine environment and cellular interactions. For example, MDSCs induce Th17 differentiation, mediate crosstalk between macrophages and tumor cells, and reshape the local microenvironment to favor tumor cell growth and metastasis (74). They also secrete IL-10 and TGF-β to promote regulatory T cell expansion, and enhance Treg activation through arginine metabolism and TGF-β–mediated pathways, contributing to immune suppression (75, 76). Additionally, MDSCs downregulate NK cell activation by producing TGF-β and hydrogen peroxide, which suppress the expression of NK cell-activating receptors such as NKG2D, NKp46, and NKp44 (76). Some MDSC subsets, particularly under hypoxic conditions, upregulate PD-L1 expression via HIF-1α activation; however, this phenomenon is not universal across all MDSC populations (77). In summary, MDSC accumulation in the breast cancer microenvironment may compromise surgical and chemotherapeutic efficacy. Targeting MDSC recruitment and immunosuppressive functions via multiple regulatory pathways holds promise for enhancing therapeutic outcomes.

### 2.4 TAMs mediate broad immunosuppressive effects through multiple mechanisms

**Figure 1:**
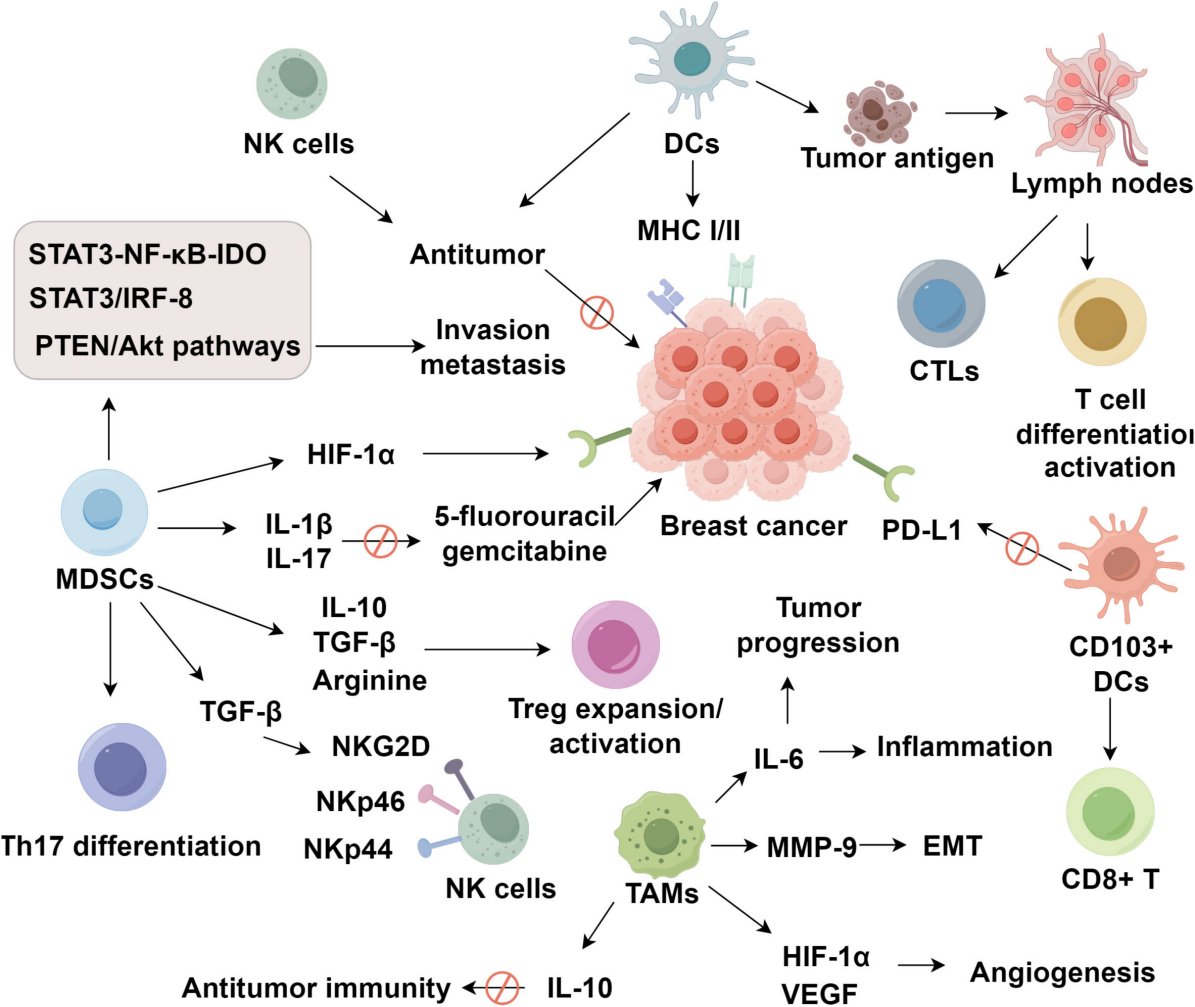
Roles of innate immune cells and cytokines in shaping the breast cancer microenvironment.

Tumor-associated macrophages (TAMs) originate from circulating monocytes that infiltrate the tumor microenvironment and subsequently undergo polarization into either classically activated M1 or alternatively activated M2 phenotypes (78). The recruitment of TAMs is driven by chemokines such as CCL2 and cytokines like CSF-1 and VEGF, which establish a permissive environment for macrophage infiltration (79–81). Once recruited, macrophage polarization is largely dictated by local signals. Hypoxic conditions, IL-4, IL-10, and TGF-β collectively promote the differentiation of macrophages into the M2 phenotype, which is closely associated with immunosuppressive and tumor-promoting functions (82–85). M2-polarized TAMs play key roles in tumor angiogenesis, epithelial–mesenchymal transition (EMT), metastasis, and tissue remodeling (86). The S1PR1 gene in TAMs inhibits pulmonary metastasis and lymphangiogenesis in murine breast cancer models by downregulating inflammatory component NLRP3 (87). COX2^+^ TAMs induce MMP-9 expression and promote EMT in the breast tumor microenvironment. The COX2/PGE2 axis also enhances IL-6 secretion from macrophages, exacerbating inflammation and further promoting tumor progression (88). Under hypoxic conditions, TAMs upregulate VEGF and HIF-1α expression to stimulate tumor angiogenesis (89). In triple-negative breast cancer, TAMs are recruited from peripheral circulation and, upon classical or alternative activation, contribute to tumor progression by suppressing cytokine production, impairing TILs function, promoting Treg expansion, and modulating PD-1 expression in the tumor milieu (90). Regarding chemoresistance, paclitaxel efficacy has been linked to M2 TAM depletion (91). Moreover, TAM-induced resistance is mediated by increased expression of Bcl-2 and STAT3, enhancing IL-10 secretion via the IL-10/STAT3/Bcl-2 signaling cascade to suppress antitumor immunity (85). In conclusion, TAMs exert multifaceted immunosuppressive effects within the breast cancer microenvironment by promoting angiogenesis, metastasis, immune evasion, and therapeutic resistance (**Figure 1**).

## 3 Innate immune factors in the tumor immune microenvironment of breast cancer

### 3.1 Dual roles of interleukins in breast cancer

Interleukins comprise a diverse family of lymphokines with pleiotropic biological activities. Their roles in breast cancer are highly context-dependent and vary by subtype. IL-6, for instance, is upregulated in over half of breast cancer patients, with elevated levels particularly noted in early-stage or high-grade tumors (92). In estrogen receptor (ER) positive breast cancer cell lines, IL-6 generally exhibits tumor-suppressive properties, while no significant effect has been observed in ER-negative cell lines (93, 94). Mechanistically, IL-6 promotes phosphorylation of JAK/STAT3 via interaction with its homodimeric or heterodimeric receptor complexes, thereby activating transcriptional programs. The feedback loop further enhances IL-6 expression via activated STAT3, rendering IL-6 less effective in breast cancer cells with low STAT3 expression (95). IL-8, a proinflammatory chemokine-like cytokine, is associated with poor survival in ER-negative patients (96). It promotes lymph node metastasis and is elevated in advanced-stage tumors. Nonetheless, some studies suggest its involvement in immune activation under certain conditions, illustrating its duality (97–99). IL-10 is generally considered an immunosuppressive cytokine and is associated with poor prognosis in breast cancer. Its expression is regulated primarily through the STAT3 and SOCS3 pathways, where STAT3 silencing markedly reduces IL-10 levels, while SOCS3 silencing enhances its expression (100). The IL-10/STAT3/Bcl-2 axis plays a pivotal role in mediating TAM-induced breast cancer cell survival and paclitaxel resistance. Inhibition of IL-10 receptor signaling enhances CD8^+^ T cell responses and upregulates IL-12 and intratumoral dendritic cells, thereby improving chemotherapy efficacy (101).

IL-11 exerts its effects through binding to IL-11 receptor alpha (IL-11Ra) and gp130, activating JAK kinases and downstream STAT3 and SOCS3. These pathways regulate tumor cell proliferation, survival, motility, and invasion (102). In breast cancer patients with bone metastases, elevated IL-11 mRNA and increased expression of p38, p-c-Jun, and p-STAT3 have been observed, highlighting its predictive value for bone metastatic potential (103). IL-15 primarily exerts indirect antitumor effects in the TIME. Through activation of PI3K signaling, IL-15 selectively stimulates T lymphocytes and enhances vaccine-like antitumor responses in combination with MEK inhibitors (104). It also potentiates NK cell-mediated cytotoxicity against CD44^+^CD24^-^ breast cancer stem-like cells and augments cetuximab efficacy (105). In a study by Gillgrass et al. (106), C57BL/6 mice receiving IL-15 via intravenous injection or harboring IL-15 transgenes exhibited a tenfold reduction in breast cancer metastasis compared to controls, likely due to enhanced NK cell cytotoxicity. Collectively, ILs exert tumor-promoting or tumor-suppressing effects in breast cancer primarily through engagement with specific receptors and activation of downstream signaling cascades. Deciphering their mechanistic roles may offer prognostic biomarkers and therapeutic targets (**Supplementary Table S1**).

### 3.2 Chemokines promote breast cancer cell invasion and metastasis

A growing body of clinical evidence supports the pivotal role of chemokines in breast cancer metastatic dissemination, and prognosis (107, 108). CCL2 and CCL5 are among the most extensively studied chemokines in the breast cancer microenvironment. In estrogen-rich conditions, both enhance tumor cell dissemination (109). Notably, levels of CCL2 and CCL5 are significantly elevated in the blood of breast cancer patients, particularly those with ER-positive tumors, and positively correlate with TAM infiltration (110). Persistent expression of CCL2 by mammary epithelial cells promotes chronic low-grade inflammation, increases glandular density, and elevates cancer risk (111). CCL2 may also modulate monocyte–macrophage crosstalk within the tumor niche (112). ELISA results indicate genotype-dependent differences in CCL2 expression across breast cancer cell suspensions, with an inverse correlation to ER and PR status. Kaplan–Meier analysis further associates low CCL2 levels with favorable prognosis (112). CCL5 enhances GLUT1 expression on tumor cells, promoting glucose uptake and metabolic reprogramming to support proliferation (113). In CCL5-deficient mice, both primary tumor burden and pulmonary metastases are markedly reduced. This may be attributed to CCR3 activation, Gfi1 expression, and Th2 polarization, which collectively establish a pre-metastatic niche conducive to myeloid cell recruitment (114). CCL18, CCL20, and CCL25 similarly contribute to prognosis prediction and promote TAM infiltration, angiogenesis, and metastatic progression (115–117). Serum CCL18 levels are significantly higher in breast cancer patients than in those with benign tumors or healthy controls, correlating with advanced clinical stage and poor survival (118). CCL20 facilitates tumor invasion and MMP-2/9 secretion in basal-like TNBC, with high CCL20 expression predicting reduced metastasis-free and overall survival (119). CCL25 promotes EMT via the CCL25/CCR9 axis, enhancing invasiveness and metastatic potential (120). Besides, CXCL1 expression in the tumor stroma is associated with tumor grade and recurrence, likely due to its negative regulation by TGF-β (121). CXCL13 transcription correlates with pathological complete response rates and favorable immune responses in breast cancer, possibly through activation of TFH13 cells and differentiation of germinal center memory B cells, thereby shifting from regulatory T cell–mediated suppression toward effective humoral immunity (122, 123).

### 3.3 TNF-α: a double-edged sword in breast cancer progression

TNF-α, a proinflammatory cytokine, orchestrates tissue homeostasis by regulating cytokine production, cell survival, and apoptosis (124). On one hand, TNF-α induces cell cycle arrest in ER-positive breast cancer cells at the G0/G1 phase, impeding DNA synthesis and exerting tumor-suppressive effects. On the other hand, it activates the NF-κB pathway and facilitates RIP1 ubiquitination, thereby stimulating JNK/ROS signaling and promoting tumor cell proliferation, enhancing the cytotoxic effects of chemotherapy and radiotherapy both *in vitro* and *in vivo* (125). Clinical data suggest that TNF-α levels are negative associated with breast cancer progression risk (126). However, TNF-α can also promote tumor growth, migration, and invasion, potentially through activation of the Wnt pathway and establishment of a tumor-permissive niche (127). Notably, the interpretation of TNF-α’s dual roles is limited by heterogeneity in immune status, inflammation levels, and disease stage across studies, necessitating further investigation (128).

### 3.4 TGF-β promotes breast cancer cell proliferation and metastasis

TGF-β, primarily synthesized by platelets, monocytes/macrophages, lymphocytes, fibroblasts, and epithelial cells, plays a central role in tumor progression, with TGF-β1 as the predominant isoform (129). TGF-β1 stimulates angiogenesis and enhances tumor cell affinity, invasiveness, and adhesion, while inhibiting normal mammary epithelial cell proliferation (130). Current research implicates the Smad signaling pathway as a major mediator of TGF-β-induced distant metastasis in breast cancer (131). TGF-β also suppresses IL-2 production and impairs T cell antitumor activity (132, 133). Additionally, it upregulates local cytokine expression, activates infiltrating immune cells, inhibits granzyme and perforin expression, downregulates MHC class I on tumor cells, and diminishes NK cell–mediated cytotoxicity (134). Importantly, TGF-β signaling has been shown to facilitate EMT and endow breast cancer cells with stem cell–like properties (135, 136). This dual role not only promotes tumor invasion and metastasis but also contributes to immune evasion through induction of an immunosuppressive microenvironment. Mechanistically, TGF-β activates EMT through canonical Smad-mediated transcriptional reprogramming and MAPK signaling pathways (137). Recent studies demonstrate that FAP/VCAN enhances the expression of EMT-associated transcription factors, further driving mesenchymal transition and increasing tumor cell plasticity (138). Moreover, TGF-β–induced PI3K/Akt activation promotes the expression of stemness markers like ALDH1 and CD44^high/CD24^low, enabling tumor-initiating capacity and resistance to chemotherapy (139). This signaling crosstalk between PI3K/Akt and Smad pathways orchestrates both immune suppression and cellular reprogramming, allowing breast cancer cells to evade immune surveillance while acquiring aggressive phenotypes. Through PI3K/Akt signaling, TGF-β can further induce EMT, thereby enhancing tumor growth and dissemination (140, 141).

## 4 Conclusion

The breast cancer immune microenvironment is profoundly shaped by the dual roles of innate immune cells and cytokines, which can either support antitumor immunity or promote immune evasion and disease progression. MDSCs and TAMs are central mediators of immunosuppression through pathways such as STAT3/NF-κB and IL-10/STAT3/Bcl-2, while NK cells and dendritic cells retain critical, yet often impaired, antitumor functions. Additionally, cytokines such as IL-6, IL-8, IL-10, TNF-α, and TGF-β demonstrate context-dependent activities, intricately regulating immune responses, tumor growth, and metastasis. The functional plasticity of these innate components highlights both the complexity and therapeutic potential of targeting the innate immune axis in breast cancer. Particularly in subtypes like triple-negative breast cancer, which lack effective targeted therapies, strategies aimed at reprogramming innate immune cells, blocking suppressive cytokines, or restoring cytotoxic activity could significantly enhance clinical outcomes. A deeper mechanistic understanding of innate immunity will not only advance prognostic biomarker development but also enable the design of rational combination therapies that synergize immunomodulation with conventional and emerging treatment modalities.

## References

[1] Oliveira FP Nogueira ML Galvão AFC Dias RB Bezerra DP. Translational drugs targeting cancer stem cells in triple-negative breast cancer. Mol Ther Oncol. (2025) 33:201008. doi: 10.1016/j.omton.2025.201008, PMID: 40687439 PMC12269295

[2] Xie H Xi X Lei T Liu H Xia Z. CD8(+) T cell exhaustion in the tumor microenvironment of breast cancer. Front Immunol. (2024) 15:1507283. doi: 10.3389/fimmu.2024.1507283, PMID: 39717767 PMC11663851

[3] Yan J Ye G Jin Y Miao M Li Q Zhou H. Identification of novel prognostic circRNA biomarkers in circRNA-miRNA-mRNA regulatory network in gastric cancer and immune infiltration analysis. BMC Genomics. (2023) 24:323. doi: 10.1186/s12864-023-09421-2, PMID: 37312060 PMC10262520

[4] Zhang Y Qin N Wang X Liang R Liu Q Geng R. Glycogen metabolism-mediated intercellular communication in the tumor microenvironment influences liver cancer prognosis. Oncol Res. (2024) 32:563–76. doi: 10.32604/or.2023.029697, PMID: 38361757 PMC10865732

[5] Wan M Mei J Cai Y Zhou J Xue N Jiang Y. Targeting IGF1R overcomes armored and cold tumor microenvironment and boosts immune checkpoint blockade in triple-negative breast cancer. Adv Sci (Weinh). (2025) 2025:e01341. doi: 10.1002/advs.202501341, PMID: 40679093 PMC12533147

[6] Tsutsumi E Macy AM LoBello J Hastings KT Kim S. Tumor immune microenvironment permissive to metastatic progression of ING4-deficient breast cancer. PloS One. (2024) 19:e0304194. doi: 10.1371/journal.pone.0304194, PMID: 38968186 PMC11226078

[7] Wang X Tan B Liu J Wang J Chen M Yang Q. EChinacoside inhibits tumor immune evasion by downregulating inducible PD-L1 and reshaping tumor immune landscape in breast and colorectal cancer. Phytomedicine. (2024) 135:156188. doi: 10.1016/j.phymed.2024.156188, PMID: 39488102

[8] Wang X Jian Q Zhang Z Gu J Wang X Wang Y. Effect of tumor-derived extracellular vesicle-shuttled lncRNA MALAT1 on proliferation, invasion and metastasis of triple-negative breast cancer by regulating macrophage M2 polarization via the POSTN/Hippo/YAP axis. Transl Oncol. (2024) 49:102076. doi: 10.1016/j.tranon.2024.102076, PMID: 39222611 PMC11402314

[9] Vallega KA Bosco DB Ren Y Sang QA. Macrophage-conditioned media promotes adipocyte cancer association, which in turn stimulates breast cancer proliferation and migration. Biomolecules. (2022) 12:1757. doi: 10.3390/biom12121757, PMID: 36551185 PMC9775594

[10] Zhang Y Yang H Jiang Y Jiang Y Mao R. Angiogenesis and immune microenvironment in triple-negative breast cancer: Targeted therapy. Biochim Biophys Acta Mol Basis Dis. (2025) 1871:167880. doi: 10.1016/j.bbadis.2025.167880, PMID: 40316057

[11] Mazloumi Z Rafat A Dizaji Asl K Nozad Charoudeh H. A combination of telomerase inhibition and NK cell therapy increased breast cancer cell line apoptosis. Biochem Biophys Res Commun. (2023) 640:50–5. doi: 10.1016/j.bbrc.2022.11.090, PMID: 36502631

[12] Imani S Farghadani R Roozitalab G Maghsoudloo M Emadi M Moradi A. Reprogramming the breast tumor immune microenvironment: cold-to-hot transition for enhanced immunotherapy. J Exp Clin Cancer Res. (2025) 44:131. doi: 10.1186/s13046-025-03394-8, PMID: 40281554 PMC12032666

[13] Miller KD O’Connor S Pniewski KA Kannan T Acosta R Mirji G. Acetate acts as a metabolic immunomodulator by bolstering T-cell effector function and potentiating antitumor immunity in breast cancer. Nat Cancer. (2023) 4:1491–507. doi: 10.1038/s43018-023-00636-6, PMID: 37723305 PMC10615731

[14] Deng Y Shi M Yi L Naveed Khan M Xia Z Li X. : Eliminating a barrier: Aiming at VISTA, reversing MDSC-mediated T cell suppression in the tumor microenvironment. Heliyon. (2024) 10:e37060. doi: 10.1016/j.heliyon.2024.e37060, PMID: 39286218 PMC11402941

[15] Tommasi C Pellegrino B Diana A Palafox Sancez M Orditura M Scartozzi M. The innate immune microenvironment in metastatic breast cancer. J Clin Med. (2022) 11:5986. doi: 10.3390/jcm11205986, PMID: 36294305 PMC9604853

[16] Mehta AK Kadel S Townsend MG Oliwa M Guerriero JL. Macrophage biology and mechanisms of immune suppression in breast cancer. Front Immunol. (2021) 12:643771. doi: 10.3389/fimmu.2021.643771, PMID: 33968034 PMC8102870

[17] Ye Y Xu C Chen F Liu Q Cheng N. Targeting innate immunity in breast cancer therapy: A narrative review. Front Immunol. (2021) 12:771201. doi: 10.3389/fimmu.2021.771201, PMID: 34899721 PMC8656691

[18] Harris MA Savas P Virassamy B O’Malley MMR Kay J Mueller SN. Towards targeting the breast cancer immune microenvironment. Nat Rev Cancer. (2024) 24:554–77. doi: 10.1038/s41568-024-00714-6, PMID: 38969810

[19] Ben-Shmuel A Gruper Y Halperin C Levi-Galibov O Rosenberg-Fogler H Barki D. Cancer-associated fibroblasts serve as decoys to suppress NK cell anticancer cytotoxicity in breast cancer. Cancer Discov. (2025) 15:1247–69. doi: 10.1158/2159-8290.CD-24-0131, PMID: 40052789

[20] Bushnell GG Sharma D Wilmot HC Zheng M Fashina TD Hutchens CM. Natural killer cell regulation of breast cancer stem cells mediates metastatic dormancy. Cancer Res. (2024) 84:3337–53. doi: 10.1158/0008-5472.CAN-24-0030, PMID: 39106452 PMC11474167

[21] Noh KM Jangid AK Park J Kim S Kim K. Membrane-immobilized gemcitabine for cancer-targetable NK cell surface engineering. J Mater Chem B. (2024) 12:12087–102. doi: 10.1039/D4TB01639D, PMID: 39465499

[22] Van der Meer JMR de Jonge P van der Waart AB Geerlings AC Moonen JP Brummelman J. CD34(+) progenitor-derived NK cell and gemcitabine combination therapy increases killing of ovarian cancer cells in NOD/SCID/IL2Rg(null) mice. Oncoimmunology. (2021) 10:1981049. doi: 10.1080/2162402X.2021.1981049, PMID: 34616589 PMC8489932

[23] Gebremeskel S Lobert L Tanner K Walker B Oliphant T Clarke LE. Natural killer T-cell immunotherapy in combination with chemotherapy-induced immunogenic cell death targets metastatic breast cancer. Cancer Immunol Res. (2017) 5:1086–97. doi: 10.1158/2326-6066.CIR-17-0229, PMID: 29054890

[24] Muraro E Comaro E Talamini R Turchet E Miolo G Scalone S. Improved Natural Killer cell activity and retained anti-tumor CD8(+) T cell responses contribute to the induction of a pathological complete response in HER2-positive breast cancer patients undergoing neoadjuvant chemotherapy. J Transl Med. (2015) 13:204. doi: 10.1186/s12967-015-0567-0, PMID: 26116238 PMC4483222

[25] Thomas N Foukakis T Willard-Gallo K. The interplay between the immune response and neoadjuvant therapy in breast cancer. Front Oncol. (2025) 15:1469982. doi: 10.3389/fonc.2025.1469982, PMID: 40421087 PMC12104209

[26] Luque M Sanz-Álvarez M Morales-Gallego M Madoz-Gúrpide J Zazo S Domínguez C. Tumor-infiltrating lymphocytes and immune response in HER2-positive breast cancer. Cancers (Basel). (2022) 14:6034. doi: 10.3390/cancers14246034, PMID: 36551522 PMC9776701

[27] Bushnell GG Sharma D Wilmot HC Zheng M Fashina TD Hutchens CM. Natural killer cell regulation of breast cancer stem cells mediates metastatic dormancy. bioRxiv. (2023) 84:3337–53. doi: 10.1101/2023.10.02.560493, PMID: 39106452 PMC11474167

[28] Neo SY Tong L Chong J Liu Y Jing X Oliveira MMS. Tumor-associated NK cells drive MDSC-mediated tumor immune tolerance through the IL-6/STAT3 axis. Sci Transl Med. (2024) 16:eadi2952. doi: 10.1126/scitranslmed.adi2952, PMID: 38748775

[29] Mamessier E Pradel LC Thibult ML Drevet C Zouine A Jacquemier J. Peripheral blood NK cells from breast cancer patients are tumor-induced composite subsets. J Immunol. (2013) 190:2424–36. doi: 10.4049/jimmunol.1200140, PMID: 23359508

[30] Rathore AS Goel MM Makker A Kumar S Srivastava AN. Is the tumor infiltrating natural killer cell (NK-TILs) count in infiltrating ductal carcinoma of breast prognostically significant? Asian Pac J Cancer Prev. (2014) 15:3757–61. doi: 10.7314/apjcp.2014.15.8.3757, PMID: 24870789

[31] Kos K Aslam MA van de Ven R Wellenstein MD Pieters W van Weverwijk A. Tumor-educated T(regs) drive organ-specific metastasis in breast cancer by impairing NK cells in the lymph node niche. Cell Rep. (2022) 38:110447. doi: 10.1016/j.celrep.2022.110447, PMID: 35235800

[32] Arianfar E Khandoozi SR Mohammadi S Memarian A. Suppression of CD56(bright) NK cells in breast cancer patients is associated with the PD-1 and TGF-βRII expression. Clin Transl Oncol. (2023) 25:841–51. doi: 10.1007/s12094-022-02997-3, PMID: 36414921

[33] Wang J Liu K Xiao T Liu P Prinz RA Xu X. Uric acid accumulation in DNA-damaged tumor cells induces NKG2D ligand expression and antitumor immunity by activating TGF-β-activated kinase 1. Oncoimmunology. (2022) 11:2016159. doi: 10.1080/2162402X.2021.2016159, PMID: 35154904 PMC8837239

[34] Lee YS Choi H Cho HR Son WC Park YS Kang CD. Downregulation of NKG2DLs by TGF-β in human lung cancer cells. BMC Immunol. (2021) 22:44. doi: 10.1186/s12865-021-00434-8, PMID: 34253166 PMC8273967

[35] Castriconi R Cantoni C Della Chiesa M Vitale M Marcenaro E Conte R. Transforming growth factor beta 1 inhibits expression of NKp30 and NKG2D receptors: consequences for the NK-mediated killing of dendritic cells. Proc Natl Acad Sci U.S.A. (2003) 100:4120–5. doi: 10.1073/pnas.0730640100, PMID: 12646700 PMC153058

[36] Hawke LG Mitchell BZ Ormiston ML. TGF-β and IL-15 synergize through MAPK pathways to drive the conversion of human NK cells to an innate lymphoid cell 1-like phenotype. J Immunol. (2020) 204:3171–81. doi: 10.4049/jimmunol.1900866, PMID: 32332109

[37] Park SI Soki FN McCauley LK. Roles of bone marrow cells in skeletal metastases: no longer bystanders. Cancer Microenviron. (2011) 4:237–46. doi: 10.1007/s12307-011-0081-8, PMID: 21809058 PMC3234319

[38] Ohl K Tenbrock K. Reactive oxygen species as regulators of MDSC-mediated immune suppression. Front Immunol. (2018) 9:2499. doi: 10.3389/fimmu.2018.02499, PMID: 30425715 PMC6218613

[39] Zhang H Wang J Li F. Modulation of natural killer cell exhaustion in the lungs: the key components from lung microenvironment and lung tumor microenvironment. Front Immunol. (2023) 14:1286986. doi: 10.3389/fimmu.2023.1286986, PMID: 38022613 PMC10657845

[40] Sun H Huang Q Huang M Wen H Lin R Zheng M. Human CD96 correlates to natural killer cell exhaustion and predicts the prognosis of human hepatocellular carcinoma. Hepatology. (2019) 70:168–83. doi: 10.1002/hep.30347, PMID: 30411378

[41] Manoury B Maisonneuve L Podsypanina K. The role of endoplasmic reticulum stress in the MHC class I antigen presentation pathway of dendritic cells. Mol Immunol. (2022) 144:44–8. doi: 10.1016/j.molimm.2022.02.007, PMID: 35184022

[42] Chatterjee F Spranger S. MHC-dressing on dendritic cells: Boosting anti-tumor immunity via unconventional tumor antigen presentation. Semin Immunol. (2023) 66:101710. doi: 10.1016/j.smim.2023.101710, PMID: 36640616

[43] Shevchenko JA Khristin AA Kurilin VV Kuznetsova MS Blinova DD Starostina NM. Autologous dendritic cells and activated cytotoxic T−cells as combination therapy for breast cancer. Oncol Rep. (2020) 43:671–80. doi: 10.3892/or.2019.7435, PMID: 31894312

[44] Luangwattananun P Chiraphapphaiboon W Thuwajit C Junking M Yenchitsomanus PT. Activation of cytotoxic T lymphocytes by self-differentiated myeloid-derived dendritic cells for killing breast cancer cells expressing folate receptor alpha protein. Bioengineered. (2022) 13:14188–203. doi: 10.1080/21655979.2022.2084262, PMID: 35734827 PMC9342379

[45] Safaei S Alipour S Bahojb Mahdavi SZ Shalmashi H Shahgoli VK Shanehbandi D. Triple-negative breast cancer-derived exosomes change the immunological features of human monocyte-derived dendritic cells and influence T-cell responses. Mol Biol Rep. (2024) 51:1058. doi: 10.1007/s11033-024-10007-8, PMID: 39417912

[46] de Mingo Pulido Á Gardner A Hiebler S Soliman H Rugo HS Krummel MF. TIM-3 regulates CD103(+) dendritic cell function and response to chemotherapy in breast cancer. Cancer Cell. (2018) 33:60–74.e66. doi: 10.1016/j.ccell.2017.11.019, PMID: 29316433 PMC5764109

[47] Borges TJ Murakami N MaChado FD Murshid A Lang BJ Lopes RL. March1-dependent modulation of donor MHC II on CD103(+) dendritic cells mitigates alloimmunity. Nat Commun. (2018) 9:3482. doi: 10.1038/s41467-018-05572-z, PMID: 30154416 PMC6113260

[48] Montealegre S van Endert P. MHC class I cross-presentation: stage lights on sec22b. Trends Immunol. (2017) 38:618–21. doi: 10.1016/j.it.2017.07.002, PMID: 28743621

[49] Patel R Sad S. Transcription factor Batf3 is important for development of CD8+ T-cell response against a phagosomal bacterium regardless of the location of antigen. Immunol Cell Biol. (2016) 94:378–87. doi: 10.1038/icb.2015.98, PMID: 26567886

[50] Vatner RE Janssen EM. STING, DCs and the link between innate and adaptive tumor immunity. Mol Immunol. (2019) 110:13–23. doi: 10.1016/j.molimm.2017.12.001, PMID: 29273394 PMC6768428

[51] Zhou Y Slone N Chrisikos TT Kyrysyuk O Babcock RL Medik YB. Vaccine efficacy against primary and metastatic cancer with *in vitro*-generated CD103(+) conventional dendritic cells. J Immunother Cancer. (2020) 8:e000474. doi: 10.1136/jitc-2019-000474, PMID: 32273347 PMC7254126

[52] Hong Y Kim YK Kim GB Nam GH Kim SA Park Y. Degradation of tumour stromal hyaluronan by small extracellular vesicle-PH20 stimulates CD103(+) dendritic cells and in combination with PD-L1 blockade boosts anti-tumour immunity. J Extracell Vesicles. (2019) 8:1670893. doi: 10.1080/20013078.2019.1670893, PMID: 31632619 PMC6781230

[53] Wu TC Xu K Banchereau R Marches F Yu CI Martinek J. Reprogramming tumor-infiltrating dendritic cells for CD103+ CD8+ mucosal T-cell differentiation and breast cancer rejection. Cancer Immunol Res. (2014) 2:487–500. doi: 10.1158/2326-6066.CIR-13-0217, PMID: 24795361 PMC4014008

[54] Chrisikos TT Zhou Y Li HS Babcock RL Wan X Patel B. STAT3 inhibits CD103(+) cDC1 vaccine efficacy in murine breast cancer. Cancers (Basel). (2020) 12:128. doi: 10.3390/cancers12010128, PMID: 31947933 PMC7017236

[55] Shigehiro T Ueno M Kijihira M Takahashi R Umemura C Taha EA. Immune state conversion of the mesenteric lymph node in a mouse breast cancer model. Int J Mol Sci. (2022) 23:11035. doi: 10.3390/ijms231911035, PMID: 36232335 PMC9570492

[56] Salmon H Idoyaga J Rahman A Leboeuf M Remark R Jordan S. Expansion and activation of CD103(+) dendritic cell progenitors at the tumor site enhances tumor responses to therapeutic PD-L1 and BRAF inhibition. Immunity. (2016) 44:924–38. doi: 10.1016/j.immuni.2016.03.012, PMID: 27096321 PMC4980762

[57] Wesolowski R Duggan MC Stiff A Markowitz J Trikha P Levine KM. 3rd: Circulating myeloid-derived suppressor cells increase in patients undergoing neo-adjuvant chemotherapy for breast cancer. Cancer Immunol Immunother. (2017) 66:1437–47. doi: 10.1007/s00262-017-2038-3, PMID: 28688082 PMC5647220

[58] Zhang R Dong M Tu J Li F Deng Q Xu J. PMN-MDSCs modulated by CCL20 from cancer cells promoted breast cancer cell stemness through CXCL2-CXCR2 pathway. Signal Transduct Target Ther. (2023) 8:97. doi: 10.1038/s41392-023-01337-3, PMID: 36859354 PMC9977784

[59] Bergenfelz C Larsson AM von Stedingk K Gruvberger-Saal S Aaltonen K Jansson S. Systemic monocytic-MDSCs are generated from monocytes and correlate with disease progression in breast cancer patients. PloS One. (2015) 10:e0127028. doi: 10.1371/journal.pone.0127028, PMID: 25992611 PMC4439153

[60] Ornstein MC Diaz-Montero CM Rayman P Elson P Haywood S Finke JH. Myeloid-derived suppressors cells (MDSC) correlate with clinicopathologic factors and pathologic complete response (pCR) in patients with urothelial carcinoma (UC) undergoing cystectomy. Urol Oncol. (2018) 36:405–12. doi: 10.1016/j.urolonc.2018.02.018, PMID: 29606341

[61] Montero AJ Diaz-Montero CM Deutsch YE Hurley J Koniaris LG Rumboldt T. Phase 2 study of neoadjuvant treatment with NOV-002 in combination with doxorubicin and cyclophosphamide followed by docetaxel in patients with HER-2 negative clinical stage II-IIIc breast cancer. Breast Cancer Res Treat. (2012) 132:215–23. doi: 10.1007/s10549-011-1889-0, PMID: 22138748 PMC3697917

[62] Solito S Falisi E Diaz-Montero CM Doni A Pinton L Rosato A. A human promyelocytic-like population is responsible for the immune suppression mediated by myeloid-derived suppressor cells. Blood. (2011) 118:2254–65. doi: 10.1182/blood-2010-12-325753, PMID: 21734236 PMC3709641

[63] Liu H Wang Z Zhou Y Yang Y. MDSCs in breast cancer: an important enabler of tumor progression and an emerging therapeutic target. Front Immunol. (2023) 14:1199273. doi: 10.3389/fimmu.2023.1199273, PMID: 37465670 PMC10350567

[64] Bruchard M Mignot G Derangère V Chalmin F Chevriaux A Végran F. Chemotherapy-triggered cathepsin B release in myeloid-derived suppressor cells activates the Nlrp3 inflammasome and promotes tumor growth. Nat Med. (2013) 19:57–64. doi: 10.1038/nm.2999, PMID: 23202296

[65] Shou D Wen L Song Z Yin J Sun Q Gong W. Suppressive role of myeloid-derived suppressor cells (MDSCs) in the microenvironment of breast cancer and targeted immunotherapies. Oncotarget. (2016) 7:64505–11. doi: 10.18632/oncotarget.11352, PMID: 27542274 PMC5325458

[66] Mao D Feng L Gong H. The antitumor and immunomodulatory effect of yanghe decoction in breast cancer is related to the modulation of the JAK/STAT signaling pathway. Evid Based Complement Alternat Med. (2018) 2018:8460526. doi: 10.1155/2018/8460526, PMID: 30581487 PMC6276440

[67] Dar AA Patil RS Pradhan TN Chaukar DA D’Cruz AK Chiplunkar SV. Myeloid-derived suppressor cells impede T cell functionality and promote Th17 differentiation in oral squamous cell carcinoma. Cancer Immunol Immunother. (2020) 69:1071–86. doi: 10.1007/s00262-020-02523-w, PMID: 32103293 PMC11027600

[68] Ma Z Zhen Y Hu C Yi H. Myeloid-derived suppressor cell-derived arginase-1 oppositely modulates IL-17A and IL-17F through the ESR/STAT3 pathway during colitis in mice. Front Immunol. (2020) 11:687. doi: 10.3389/fimmu.2020.00687, PMID: 32391010 PMC7188946

[69] Li X Lu P Li B Zhang W Yang R Chu Y. Interleukin 2 and interleukin 10 function synergistically to promote CD8(+) T cell cytotoxicity, which is suppressed by regulatory T cells in breast cancer. Int J Biochem Cell Biol. (2017) 87:1–7. doi: 10.1016/j.biocel.2017.03.003, PMID: 28274688 PMC7185534

[70] Lechner MG Megiel C Russell SM Bingham B Arger N Woo T. Functional characterization of human Cd33+ and Cd11b+ myeloid-derived suppressor cell subsets induced from peripheral blood mononuclear cells co-cultured with a diverse set of human tumor cell lines. J Transl Med. (2011) 9:90. doi: 10.1186/1479-5876-9-90, PMID: 21658270 PMC3128058

[71] Parker KH Beury DW Ostrand-Rosenberg S. Myeloid-derived suppressor cells: critical cells driving immune suppression in the tumor microenvironment. Adv Cancer Res. (2015) 128:95–139. doi: 10.1016/bs.acr.2015.04.002, PMID: 26216631 PMC4662416

[72] Xu W Jiang T Shen K Zhao D Zhang M Zhu W. GADD45B regulates the carcinogenesis process of chronic atrophic gastritis and the metabolic pathways of gastric cancer. Front Endocrinol (Lausanne). (2023) 14:1224832. doi: 10.3389/fendo.2023.1224832, PMID: 37608794 PMC10441793

[73] Oo MW Kawai H Takabatake K Tomida S Eguchi T Ono K. Resident stroma-secreted chemokine CCL2 governs myeloid-derived suppressor cells in the tumor microenvironment. JCI Insight. (2022) 7:e148960. doi: 10.1172/jci.insight.148960, PMID: 34874922 PMC8765046

[74] Umansky V Blattner C Gebhardt C Utikal J. The role of myeloid-derived suppressor cells (MDSC) in cancer progression. Vaccines (Basel). (2016) 4:36. doi: 10.3390/vaccines4040036, PMID: 27827871 PMC5192356

[75] Tomić S Joksimović B Bekić M Vasiljević M Milanović M Čolić M. Prostaglanin-E2 potentiates the suppressive functions of human mononuclear myeloid-derived suppressor cells and increases their capacity to expand IL-10-producing regulatory T cell subsets. Front Immunol. (2019) 10:475. doi: 10.3389/fimmu.2019.00475, PMID: 30936876 PMC6431635

[76] Najafi M Farhood B Mortezaee K. Contribution of regulatory T cells to cancer: A review. J Cell Physiol. (2019) 234:7983–93. doi: 10.1002/jcp.27553, PMID: 30317612

[77] Noman MZ Desantis G Janji B Hasmim M Karray S Dessen P. PD-L1 is a novel direct target of HIF-1α, and its blockade under hypoxia enhanced MDSC-mediated T cell activation. J Exp Med. (2014) 211:781–90. doi: 10.1084/jem.20131916, PMID: 24778419 PMC4010891

[78] Tharp KM Kersten K Maller O Timblin GA Stashko C Canale FP. Tumor-associated macrophages restrict CD8(+) T cell function through collagen deposition and metabolic reprogramming of the breast cancer microenvironment. Nat Cancer. (2024) 5:1045–62. doi: 10.1038/s43018-024-00775-4, PMID: 38831058 PMC12204312

[79] Chen X Yang M Yin J Li P Zeng S Zheng G. Tumor-associated macrophages promote epithelial-mesenchymal transition and the cancer stem cell properties in triple-negative breast cancer through CCL2/AKT/β-catenin signaling. Cell Commun Signal. (2022) 20:92. doi: 10.1186/s12964-022-00888-2, PMID: 35715860 PMC9205034

[80] Duran CL Surve CR Ye X Chen X Lin Y Harney AS. Targeting CSF-1 signaling between tumor cells and macrophages at TMEM doorways inhibits breast cancer dissemination. Oncogene. (2025). doi: 10.1038/s41388-025-03485-y, PMID: 40646332 PMC12399421

[81] Wang L Zhang L Zhao L Shao S Ning Q Jing X. VEGFA/NRP-1/GAPVD1 axis promotes progression and cancer stemness of triple-negative breast cancer by enhancing tumor cell-macrophage crosstalk. Int J Biol Sci. (2024) 20:446–63. doi: 10.7150/ijbs.86085, PMID: 38169627 PMC10758102

[82] Chen F Chen J Yang L Liu J Zhang X Zhang Y. Extracellular vesicle-packaged HIF-1α-stabilizing lncRNA from tumour-associated macrophages regulates aerobic glycolysis of breast cancer cells. Nat Cell Biol. (2019) 21:498–510. doi: 10.1038/s41556-019-0299-0, PMID: 30936474

[83] Rahal OM Wolfe AR Mandal PK Larson R Tin S Jimenez C. Blocking interleukin (IL)4- and IL13-mediated phosphorylation of STAT6 (Tyr641) decreases M2 polarization of macrophages and protects against macrophage-mediated radioresistance of inflammatory breast cancer. Int J Radiat Oncol Biol Phys. (2018) 100:1034–43. doi: 10.1016/j.ijrobp.2017.11.043, PMID: 29485045

[84] Zhang J Dong Y Yu S Hu K Zhang L Xiong M. IL-4/IL-4R axis signaling drives resistance to immunotherapy by inducing the upregulation of Fcγ receptor IIB in M2 macrophages. Cell Death Dis. (2024) 15:500. doi: 10.1038/s41419-024-06875-4, PMID: 39003253 PMC11246528

[85] Yang C He L He P Liu Y Wang W He Y. Increased drug resistance in breast cancer by tumor-associated macrophages through IL-10/STAT3/bcl-2 signaling pathway. Med Oncol. (2015) 32:352. doi: 10.1007/s12032-014-0352-6, PMID: 25572805

[86] Melwani PK Checker R Balla MMS Pandey BN. Crosstalk between macrophages and breast cancer cells: networking within tumors. Results Probl Cell Differ. (2024) 74:213–38. doi: 10.1007/978-3-031-65944-7_8, PMID: 39406907

[87] Weichand B Popp R Dziumbla S Mora J Strack E Elwakeel E. S1PR1 on tumor-associated macrophages promotes lymphangiogenesis and metastasis via NLRP3/IL-1β. J Exp Med. (2017) 214:2695–713. doi: 10.1084/jem.20160392, PMID: 28739604 PMC5584110

[88] Gan L Qiu Z Huang J Li Y Huang H Xiang T. Cyclooxygenase-2 in tumor-associated macrophages promotes metastatic potential of breast cancer cells through Akt pathway. Int J Biol Sci. (2016) 12:1533–43. doi: 10.7150/ijbs.15943, PMID: 27994517 PMC5166494

[89] Mu G Zhu Y Dong Z Shi L Deng Y Li H. Calmodulin 2 facilitates angiogenesis and metastasis of gastric cancer via STAT3/HIF-1A/VEGF-A mediated macrophage polarization. Front Oncol. (2021) 11:727306. doi: 10.3389/fonc.2021.727306, PMID: 34604066 PMC8479158

[90] Meng Z Zhang R Wu X Zhang M Jin T. PD−L1 mediates triple−negative breast cancer evolution via the regulation of TAM/M2 polarization. Int J Oncol. (2022) 61:150. doi: 10.3892/ijo.2022.5440, PMID: 36263619 PMC9591321

[91] Deswal B Bagchi U Kapoor S. Curcumin suppresses M2 macrophage-derived paclitaxel chemoresistance through inhibition of PI3K-AKT/STAT3 signaling. Anticancer Agents Med Chem. (2024) 24:146–56. doi: 10.2174/0118715206275259231105184959, PMID: 37957871

[92] Ma Y Ren Y Dai ZJ Wu CJ Ji YH Xu J. IL-6, IL-8 and TNF-α levels correlate with disease stage in breast cancer patients. Adv Clin Exp Med. (2017) 26:421–6. doi: 10.17219/acem/62120, PMID: 28791816

[93] Xing J Li J Fu L Gai J Guan J Li Q. SIRT4 enhances the sensitivity of ER-positive breast cancer to tamoxifen by inhibiting the IL-6/STAT3 signal pathway. Cancer Med. (2019) 8:7086–97. doi: 10.1002/cam4.2557, PMID: 31573734 PMC6853819

[94] Dethlefsen C Højfeldt G Hojman P. The role of intratumoral and systemic IL-6 in breast cancer. Breast Cancer Res Treat. (2013) 138:657–64. doi: 10.1007/s10549-013-2488-z, PMID: 23532539

[95] Manore SG Doheny DL Wong GL Lo HW. IL-6/JAK/STAT3 signaling in breast cancer metastasis: biology and treatment. Front Oncol. (2022) 12:866014. doi: 10.3389/fonc.2022.866014, PMID: 35371975 PMC8964978

[96] Freund A Chauveau C Brouillet JP Lucas A Lacroix M Licznar A. IL-8 expression and its possible relationship with estrogen-receptor-negative status of breast cancer cells. Oncogene. (2003) 22:256–65. doi: 10.1038/sj.onc.1206113, PMID: 12527894 PMC2034407

[97] Liao H Li H Song J Chen H Si H Dong J. Expression of the prognostic marker IL-8 correlates with the immune signature and epithelial-mesenchymal transition in breast cancer. J Clin Lab Anal. (2023) 37:e24797. doi: 10.1002/jcla.24797, PMID: 36725216 PMC9978063

[98] Dominguez C McCampbell KK David JM Palena C. Neutralization of IL-8 decreases tumor PMN-MDSCs and reduces mesenchymalization of claudin-low triple-negative breast cancer. JCI Insight. (2017) 2:e94296. doi: 10.1172/jci.insight.94296, PMID: 29093275 PMC5752275

[99] Foldi J Blenman KRM Marczyk M Gunasekharan V Polanska A Gee R. Peripheral blood immune parameters, response, and adverse events after neoadjuvant chemotherapy plus durvalumab in early-stage triple-negative breast cancer. Breast Cancer Res Treat. (2024) 208:369–77. doi: 10.1007/s10549-024-07426-3, PMID: 39002068 PMC13327071

[100] Lee EB Kim A Kang K Kim H Lim JS. NDRG2-mediated modulation of SOCS3 and STAT3 activity inhibits IL-10 production. Immune Netw. (2010) 10:219–29. doi: 10.4110/in.2010.10.6.219, PMID: 21286383 PMC3026942

[101] Ruffell B Chang-Strachan D Chan V Rosenbusch A Ho CM Pryer N. Macrophage IL-10 blocks CD8+ T cell-dependent responses to chemotherapy by suppressing IL-12 expression in intratumoral dendritic cells. Cancer Cell. (2014) 26:623–37. doi: 10.1016/j.ccell.2014.09.006, PMID: 25446896 PMC4254570

[102] Ren L Wang X Dong Z Liu J Zhang S. Bone metastasis from breast cancer involves elevated IL-11 expression and the gp130/STAT3 pathway. Med Oncol. (2013) 30:634. doi: 10.1007/s12032-013-0634-4, PMID: 23813018

[103] Johnstone CN Chand A Putoczki TL Ernst M. Emerging roles for IL-11 signaling in cancer development and progression: Focus on breast cancer. Cytokine Growth Factor Rev. (2015) 26:489–98. doi: 10.1016/j.cytogfr.2015.07.015, PMID: 26209885

[104] Allegrezza MJ Rutkowski MR Stephen TL Svoronos N Tesone AJ Perales-Puchalt A. IL15 agonists overcome the immunosuppressive effects of MEK inhibitors. Cancer Res. (2016) 76:2561–72. doi: 10.1158/0008-5472.CAN-15-2808, PMID: 26980764 PMC4873368

[105] Roberti MP Rocca YS Amat M Pampena MB Loza J Coló F. IL-2- or IL-15-activated NK cells enhance Cetuximab-mediated activity against triple-negative breast cancer in xenografts and in breast cancer patients. Breast Cancer Res Treat. (2012) 136:659–71. doi: 10.1007/s10549-012-2287-y, PMID: 23065032

[106] Gillgrass A Gill N Babian A Ashkar AA. The absence or overexpression of IL-15 drastically alters breast cancer metastasis via effects on NK cells, CD4 T cells, and macrophages. J Immunol. (2014) 193:6184–91. doi: 10.4049/jimmunol.1303175, PMID: 25355926

[107] Yoshimura T Li C Wang Y Matsukawa A. The chemokine monocyte chemoattractant protein-1/CCL2 is a promoter of breast cancer metastasis. Cell Mol Immunol. (2023) 20:714–38. doi: 10.1038/s41423-023-01013-0, PMID: 37208442 PMC10310763

[108] Valdivia-Silva J Chinney-Herrera A. Chemokine receptors and their ligands in breast cancer: The key roles in progression and metastasis. Int Rev Cell Mol Biol. (2024) 388:124–61. doi: 10.1016/bs.ircmb.2024.07.002, PMID: 39260935

[109] Li X Wang M Gong T Lei X Hu T Tian M. A S100A14-CCL2/CXCL5 signaling axis drives breast cancer metastasis. Theranostics. (2020) 10:5687–703. doi: 10.7150/thno.42087, PMID: 32483412 PMC7255008

[110] Svensson S Abrahamsson A Rodriguez GV Olsson AK Jensen L Cao Y. CCL2 and CCL5 are novel therapeutic targets for estrogen-dependent breast cancer. Clin Cancer Res. (2015) 21:3794–805. doi: 10.1158/1078-0432.CCR-15-0204, PMID: 25901081

[111] Sun X Glynn DJ Hodson LJ Huo C Britt K Thompson EW. CCL2-driven inflammation increases mammary gland stromal density and cancer susceptibility in a transgenic mouse model. Breast Cancer Res. (2017) 19:4. doi: 10.1186/s13058-016-0796-z, PMID: 28077158 PMC5225654

[112] Mandal PK Biswas S Mandal G Purohit S Gupta A Majumdar Giri A. CCL2 conditionally determines CCL22-dependent Th2-accumulation during TGF-β-induced breast cancer progression. Immunobiology. (2018) 223:151–61. doi: 10.1016/j.imbio.2017.10.031, PMID: 29107385

[113] Gao D Rahbar R Fish EN. CCL5 activation of CCR5 regulates cell metabolism to enhance proliferation of breast cancer cells. Open Biol. (2016) 6:160122. doi: 10.1098/rsob.160122, PMID: 27335323 PMC4929946

[114] Zhang Q Qin J Zhong L Gong L Zhang B Zhang Y. CCL5-mediated th2 immune polarization promotes metastasis in luminal breast cancer. Cancer Res. (2015) 75:4312–21. doi: 10.1158/0008-5472.CAN-14-3590, PMID: 26249173

[115] Zhao C Zheng S Yan Z Deng Z Wang R Zhang B. CCL18 promotes the invasion and metastasis of breast cancer through Annexin A2. Oncol Rep. (2020) 43:571–80. doi: 10.3892/or.2019.7426, PMID: 31894281

[116] Kwantwi LB Wang S Sheng Y Wu Q. Multifaceted roles of CCL20 (C-C motif chemokine ligand 20): mechanisms and communication networks in breast cancer progression. Bioengineered. (2021) 12:6923–34. doi: 10.1080/21655979.2021.1974765, PMID: 34569432 PMC8806797

[117] Chen H Cong X Wu C Wu X Wang J Mao K. Intratumoral delivery of CCL25 enhances immunotherapy against triple-negative breast cancer by recruiting CCR9(+) T cells. Sci Adv. (2020) 6:eaax4690. doi: 10.1126/sciadv.aax4690, PMID: 32064335 PMC6989134

[118] Sun JH Fan N Zhang Y. Correlation between serum level of chemokine (C-C motif) ligand 18 and poor prognosis in breast cancer. Genet Mol Res. (2016) 15. doi: 10.4238/gmr.15038632, PMID: 27706714

[119] Lee SK Park KK Kim HJ Park J Son SH Kim KR. Human antigen R-regulated CCL20 contributes to osteolytic breast cancer bone metastasis. Sci Rep. (2017) 7:9610. doi: 10.1038/s41598-017-09040-4, PMID: 28851919 PMC5575024

[120] Zhang Z Sun T Chen Y Gong S Sun X Zou F. CCL25/CCR9 signal promotes migration and invasion in hepatocellular and breast cancer cell lines. DNA Cell Biol. (2016) 35:348–57. doi: 10.1089/dna.2015.3104, PMID: 27008282

[121] Zou A Lambert D Yeh H Yasukawa K Behbod F Fan F. Elevated CXCL1 expression in breast cancer stroma predicts poor prognosis and is inversely associated with expression of TGF-β signaling proteins. BMC Cancer. (2014) 14:781. doi: 10.1186/1471-2407-14-781, PMID: 25344051 PMC4221705

[122] Gu-Trantien C Migliori E Buisseret L de Wind A Brohée S Garaud S. CXCL13-producing TFH cells link immune suppression and adaptive memory in human breast cancer. JCI Insight. (2017) 2:e91487. doi: 10.1172/jci.insight.91487, PMID: 28570278 PMC5453706

[123] Bedognetti D Wang E Marincola FM. Meta-analysis and metagenes: CXCL-13-driven signature as a robust marker of intratumoral immune response and predictor of breast cancer chemotherapeutic outcome. Oncoimmunology. (2014) 3:e28727. doi: 10.4161/onci.28727, PMID: 25340012 PMC4203509

[124] Cruceriu D Baldasici O Balacescu O Berindan-Neagoe I. The dual role of tumor necrosis factor-alpha (TNF-α) in breast cancer: molecular insights and therapeutic approaches. Cell Oncol (Dordr). (2020) 43:1–18. doi: 10.1007/s13402-019-00489-1, PMID: 31900901 PMC12990688

[125] Shen WH Zhou JH Broussard SR Freund GG Dantzer R Kelley KW. Proinflammatory cytokines block growth of breast cancer cells by impairing signals from a growth factor receptor. Cancer Res. (2002) 62:4746–56., PMID: 12183434

[126] Muenst S Läubli H Soysal SD Zippelius A Tzankov A Hoeller S. The immune system and cancer evasion strategies: therapeutic concepts. J Intern Med. (2016) 279:541–62. doi: 10.1111/joim.12470, PMID: 26748421

[127] Chen Y Wen H Zhou C Su Q Lin Y Xie Y. TNF-α derived from M2 tumor-associated macrophages promotes epithelial-mesenchymal transition and cancer stemness through the Wnt/β-catenin pathway in SMMC-7721 hepatocellular carcinoma cells. Exp Cell Res. (2019) 378:41–50. doi: 10.1016/j.yexcr.2019.03.005, PMID: 30844387

[128] Eftekhari R Esmaeili R Mirzaei R Bidad K de Lima S Ajami M. Study of the tumor microenvironment during breast cancer progression. Cancer Cell Int. (2017) 17:123. doi: 10.1186/s12935-017-0492-9, PMID: 29299026 PMC5741925

[129] Honda CK Kurozumi S Fujii T Pourquier D Khellaf L Boissiere F. Cancer-associated fibroblast spatial heterogeneity and EMILIN1 expression in the tumor microenvironment modulate TGF-β activity and CD8(+) T-cell infiltration in breast cancer. Theranostics. (2024) 14:1873–85. doi: 10.7150/thno.90627, PMID: 38505604 PMC10945331

[130] Zhang Z Lin M Wang J Yang F Yang P Liu Y. Calycosin inhibits breast cancer cell migration and invasion by suppressing EMT via BATF/TGF-β1. Aging (Albany NY). (2021) 13:16009–23. doi: 10.18632/aging.203093, PMID: 34096887 PMC8266341

[131] Luo D Zeng X Zhang S Li D Cheng Z Wang Y. Pirfenidone suppressed triple-negative breast cancer metastasis by inhibiting the activity of the TGF-β/SMAD pathway. J Cell Mol Med. (2023) 27:456–69. doi: 10.1111/jcmm.17673, PMID: 36651490 PMC9889661

[132] Meulmeester E Ten Dijke P. The dynamic roles of TGF-β in cancer. J Pathol. (2011) 223:205–18. doi: 10.1002/path.2785, PMID: 20957627

[133] Zhao Z Luo Q Liu Y Jiang K Zhou L Dai R. Multi-level integrative analysis of the roles of lncRNAs and differential mRNAs in the progression of chronic pancreatitis to pancreatic ductal adenocarcinoma. BMC Genomics. (2023) 24:101. doi: 10.1186/s12864-023-09209-4, PMID: 36879212 PMC9990329

[134] Ma D Niederkorn JY. Transforming growth factor-beta down-regulates major histocompatibility complex class I antigen expression and increases the susceptibility of uveal melanoma cells to natural killer cell-mediated cytolysis. Immunology. (1995) 86:263–9., PMID: 7490128 PMC1384005

[135] Luo W Shi Q Han M Zhang Z Reiter RJ Ashrafizadeh M. TGF-β-driven EMT in cancer progression and drug resistance. Cytokine Growth Factor Rev. (2025). doi: 10.1016/j.cytogfr.2025.05.004, PMID: 40436672

[136] Li ZX Chen JX Zheng ZJ Cai WJ Yang XB Huang YY. TGF-β1 promotes human breast cancer angiogenesis and Malignant behavior by regulating endothelial-mesenchymal transition. Front Oncol. (2022) 12:1051148. doi: 10.3389/fonc.2022.1051148, PMID: 36465358 PMC9709251

[137] Geng XQ Ma A He JZ Wang L Jia YL Shao GY. Ganoderic acid hinders renal fibrosis via suppressing the TGF-β/Smad and MAPK signaling pathways. Acta Pharmacol Sin. (2020) 41:670–7. doi: 10.1038/s41401-019-0324-7, PMID: 31804606 PMC7468553

[138] Ping Q Wang C Cheng X Zhong Y Yan R Yang M. TGF-β1 dominates stromal fibroblast-mediated EMT via the FAP/VCAN axis in bladder cancer cells. J Transl Med. (2023) 21:475. doi: 10.1186/s12967-023-04303-3, PMID: 37461061 PMC10351189

[139] Konge J Leteurtre F Goislard M Biard D Morel-Altmeyer S Vaurijoux A. Breast cancer stem cell-like cells generated during TGFβ-induced EMT are radioresistant. Oncotarget. (2018) 9:23519–31. doi: 10.18632/oncotarget.25240, PMID: 29805752 PMC5955125

[140] Lee JC Lee KM Kim DW Heo DS. Elevated TGF-beta1 secretion and down-modulation of NKG2D underlies impaired NK cytotoxicity in cancer patients. J Immunol. (2004) 172:7335–40. doi: 10.4049/jimmunol.172.12.7335, PMID: 15187109

[141] Chen L Fu H Luo Y Chen L Cheng R Zhang N. cPLA2α mediates TGF-β-induced epithelial-mesenchymal transition in breast cancer through PI3k/Akt signaling. Cell Death Dis. (2017) 8:e2728. doi: 10.1038/cddis.2017.152, PMID: 28383549 PMC5477578

